# Prevalence of methicillin-resistant *Staphylococcus aureus* (MRSA) in street-vended tomato sauces in Dhaka, Bangladesh

**DOI:** 10.1186/s13104-026-07822-6

**Published:** 2026-05-09

**Authors:** Mizbahul Karim Hemo, Md. Aminul Islam, Suvamoy Datta

**Affiliations:** 1 Advanced Molecular Genetics Laboratory, Department of Microbiology, Primeasia University, 12 Kemal Ataturk Ave, Banani, Dhaka, 1213 Bangladesh; 2 Research and Training Development Division, MicroQuest Research & Training Center (MQRTC), Dhaka, 1229 Bangladesh; 3 Advanced Molecular Lab, Department of Microbiology, NSTU Covid-19 Lab, President Abdul Hamid Medical College Hospital (PAHMCH), Karimganj, Kishoreganj, 2310 Bangladesh; 4 Laboratory (Microbiology) and Quality Division, Vitalac Dairy and Food Industries (Nutraceuticals) Ltd., Dhaka, Bangladesh

**Keywords:** Tomato sauce contamination, Methicillin-resistant *S. aureus* (MRSA), Vancomycin-resistant S. aureus (VRSA), Street-vended food, Food safety, Multidrug resistance (MDR) bacteria, Public health risk, Antimicrobial resistance (AMR)

## Abstract

**Objective:**

To assess the prevalence of *Staphylococcus aureus* and antimicrobial resistance patterns, with particular emphasis on methicillin resistance, among isolates from street-vended tomato sauces in Dhaka, Bangladesh.

**Results:**

Among 104 street-vended tomato sauce samples, 83 (79.8%; 95% Cl: 71.1–86.4%) were *S. aureus* isolates confirmed. Of these, 38 (45.8%) identified as methicillin-resistant *Staphylococcus aureus*, 5 (6.0%) vancomycin-resistant *S. aureus*, 55 (66.3%) multidrug resistance, and 2 (2.4%) extensively drug-resistant. The multiple antibiotic resistance (MAR) index ranged from 0.08 to 0.75 (mean ± SD = 0.38 ± 0.12), with 60 (72.3%) isolates exceeding the high-risk threshold (≥ 0.2). Methicillin-Resistant isolates showed significantly higher index values than susceptible isolates (*p* = 0.003). Co-resistance observed between oxacillin-cefoxitin (φ = 1.00) and meropenem-amikacin (φ = 0.96). Elevated resistance index values were more frequently observed among isolates from slum and transport-hub areas.

**Supplementary Information:**

The online version contains supplementary material available at 10.1186/s13104-026-07822-6.

## Introduction

Food safety remains a major global public health concern, with an estimated 600 million illnesses and 420,000 deaths annually attributed to contaminated food [1, 2]. Food contamination may occur at multiple stages along the farm-to-fork continuum, particularly through inadequate hygiene and unsafe handling practices [3, 4]. However, several studies have reported poor sanitary conditions among street food vendors, including inadequate hand hygiene, use of unsafe water, and contaminated utensils, leading to frequent detection of pathogenic microorganisms in ready-to-eat foods [5–8]. Tomato (*Solanum lycopersicum L.)* based sauces are widely consumed as condiments with popular street foods in Bangladesh, such as shingara, samosa, puri, fuchka, and fried snacks. Unlike freshly prepared foods, these sauces are often produced in bulk, stored at ambient temperature, and repeatedly dispensed throughout the vending period. These practices, combined with environmental exposure, create favorable conditions for microbial growth and cross-contamination. Low-cost adulterants and improper preservation may further compromise microbiological quality [9–12]. In 2024, the WHO identified *S. aureus* as a priority pathogen [13]. As a common colonizer of human skin and mucosa, it can be easily transmitted to food through contaminated hands, utensils, or environmental surfaces. Notably, enterotoxigenic strains produce heat-stable toxins capable of causing food poisoning even after cooking or reheating [3, 14]. The emergence of antimicrobial resistance in *S. aureus*, particularly methicillin-resistant *S. aureus* (MRSA), is of increasing concern due to its association with multidrug resistance and limited treatment [15–19]. Reports of vancomycin-resistant *S. aureus* (VRSA) further highlight the potential public health implications of resistant strains in the food chain [17, 20]. Although antimicrobial resistance *S. aureus* has been reported in various foods, data on its presence in street-vended tomato sauces in Bangladesh remains limited. Therefore, this study aimed to assess the prevalence of *S. aureus* and its antimicrobial resistance profiles, with emphasis on MRSA and related resistance phenotypes, in street-vended tomato sauces in Dhaka, Bangladesh.

## Materials and methods

### Study design and sampling

This study was conducted from April 1 to September 30, 2025, in Dhaka, Bangladesh. Samples were collected from major commercial, residential, and transport-associated areas, including Banani, Mirpur, Airport, Uttara, Khilkhet, and Gulshan (Supplementary data 1). A total of 104 tomato sauce samples (25 ml each) were aseptically one per vendor. Samples were randomly collected based on vendor availability and transferred into sterile containers, where standard aseptic protocols were followed [21, 22]. All samples were transported within 2-4 h under cold-chain conditions to the Department of Microbiology Laboratory, Primeasia University, Dhaka, and microbiological analyses were performed upon arrival.

### Isolation of *Staphylococcus aureus*

Each sample (1 ml) was enriched in 9 ml peptone water and incubated at 35 °C for 24 h. Aliquots were cultured on Mannitol Salt Agar and incubated at 37 °C for 24 h. Presumptive *S. aureus* colonies were identified based on mannitol fermentation and colony morphology. Confirmation was performed by Gram staining (Gram-positive cocci in clusters), catalase (positive), and coagulase tests (positive) following standard guidelines [23]. Confirmed isolates were preserved in tryptic soy broth s with 20% glycerol and at -20 °C.

### Phenotypic identification of MRSA

MRSA screening was performed using the Kirby-Bauer disk diffusion method on Mueller Hinton Agar following CLSI (2023) guidelines [24]. relies solely on phenotypic antimicrobial susceptibility testing and does not include molecular confirmation. Cefoxitin (30 µg) and Oxacillin (1 µg) disks were used for Methicillin-Resistant. According to CLSI guidelines, the *S. aureus* showing resistance to cefoxitin is classified as *Methicillin-Resistant Staphylococcus aureus*. In addition, resistance to oxacillin is considered supplementary and not used as a defining criterion for MRSA. *S. aureus* ATCC 43,300 and ATCC 25,923 were used as control strains.

### Antimicrobial susceptibility testing (AST)

Antimicrobial susceptibility testing was performed using the Kirby-Bauer disk diffusion method on MHA following CLSI M100-S33 (2023) guidelines [24]. The bacterial suspension was adjusted to a 0.5 McFarland standard turbidity and incubated at 37 °C for 24 h. Antibiotics tested included β-lactams (oxacillin-1 µg, cefoxitin-30 µg, meropenem-10 µg), macrolides (azithromycin-15 µg, erythromycin-15 µg), lincosamide (clindamycin-2 µg), aminoglycosides (gentamicin-10 µg, amikacin-30 µg), fluoroquinolone (ciprofloxacin-5 µg), ansamycin (rifampicin-5 µg), and glycopeptide (vancomycin-30 µg). Zone diameters are interpreted according to CLSI breakpoints, and Intermediate results were considered non-susceptible.

### Classification of MDR, XDR, and PDR isolates

Antibiotic resistance profiles were interpreted following Magiorakos et al. (2012) [25]. Isolates were classified as multidrug-resistant (MDR) if they were resistant to at least one agent in three or more antimicrobial categories. Isolates were considered extensively drug-resistant (XDR) if they were resistant to at least one agent in all antimicrobial categories, remaining susceptible to only one or two categories. Pan-drug-resistant (PDR) if resistant to all agents in all classes tested. The number of resistant antibiotics (R count) was recorded for each isolate.

### Multiple antibiotic resistance (MAR) index

The MAR index was calculated for each isolate using the formula:

MAR index = Number of antibiotics to which isolation is resistant/Total Number of antibiotics tested.

Following Krumperman (1983), a MAR value ≥ 0.2 indicates high risk exposure, ≥ 0.3 indicates very high exposure [26].

### Statistical analysis

Data were compiled in Microsoft Excel 2021 and analyzed using Python version 3.11 with the pandas, NumPy, SciPy, matplotlib, and seaborn libraries. Descriptive statistics (mean, median, standard deviation, and range) are used to summarize antibiotic resistance data and MAR index values. Differences in MAR index between groups were compared using the Mann-Whitney U test (for MRSA vs. MSSA) and the Kruskal-Wallis H test (for multiple sampling areas or environments). In addition, the associations between binary antibiotic-resistance traits are evaluated using the phi (φ) correlation coefficient. Visualizations (boxplots, forest plots, and heatmaps) generated in Python. All tests were two-tailed, and *p* < 0.05 was considered statistically significant.

## Results

### Prevalence of *Staphylococcus aureus* and antimicrobial resistance phenotypes

We identified the high prevalence of *Staphylococcus aureus* contamination in tomato sauces collected from street-food vendors in Dhaka. Among 104 samples, 83 (79.8%; 95% CI: 71.1–86.4%) were confirmed as *S. aureus.* Of these isolates, 38 (45.8%) were phenotypically MRSA, 55 (66.3%) exhibited MDR, while a small proportion showed VRSA, XDR phenotypes (Table 1).

These prevalence values are summarized in Table 1 and Supplementary Fig. 1.

**Table 1:** Prevalence of *S. aureus* and resistance phenotypes in tomato sauce samples from Dhaka

Parameter	Count (*n*/*N*)	%	95% CI
*S. aureus* isolation	83/104	79.8%	71.1–86.4%
MRSA	38/83	45.8%	35.5–56.5%
VRSA	5/83	6.0%	2.6–13.3%
MDR	55/83	66.3%	55.6–75.5%
XDR	2/83	2.4%	0.7–8.4%

(*n* = 104; isolates *n* = 83). Wilson 95% confidence intervals are shown. MRSA methicillin-resistant *S. aureus*; VRSA vancomycin-resistant *S. aureus*; MDR multidrug-resistant; XDR extensively drug-resistant

### Antibiotic-resistance patterns of MRSA and MSSA isolates

**Fig. 1:**
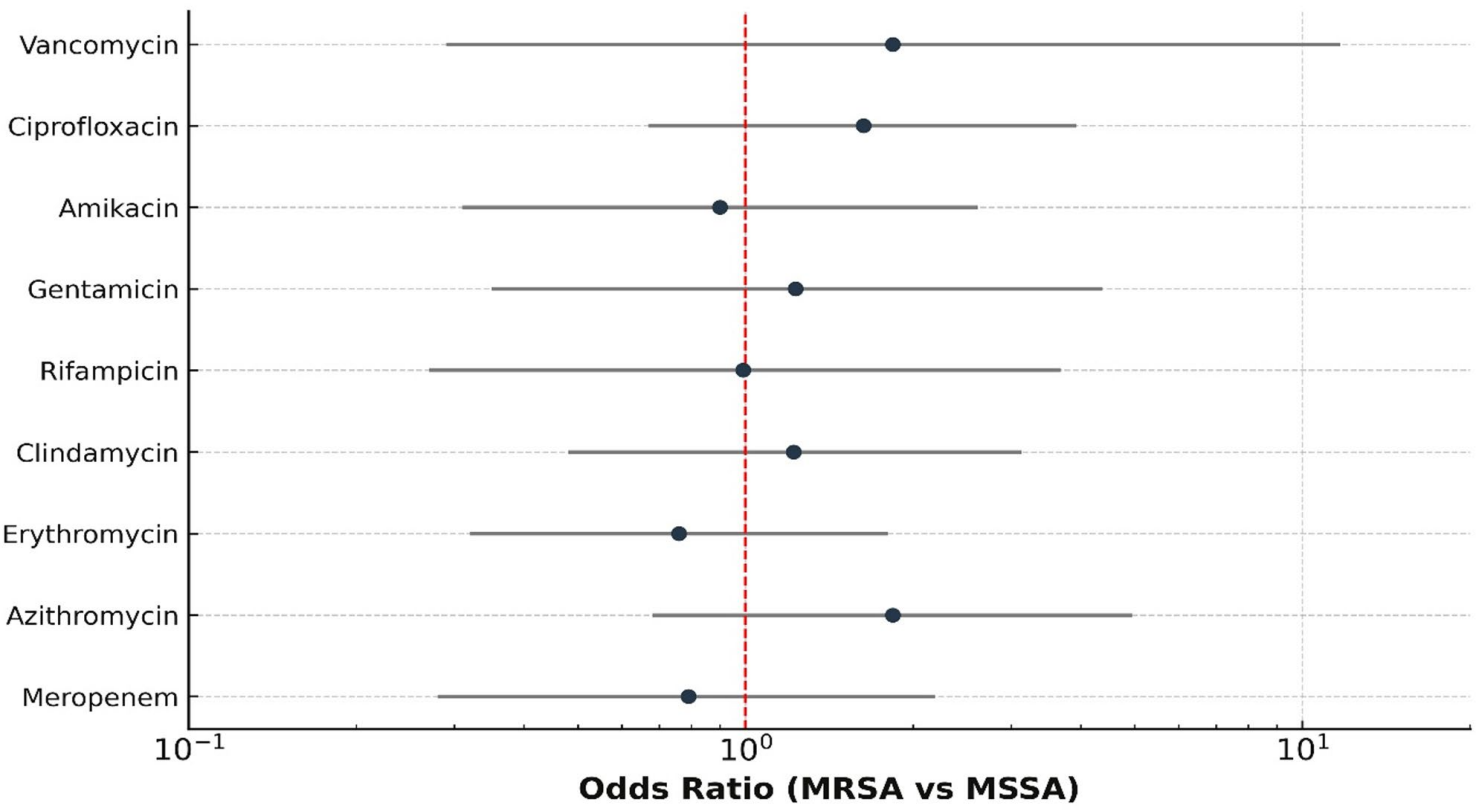
Forest plot of odds ratios (OR) with 95% confidence intervals for antibiotic resistance in MRSA compared with MSSA isolates. Each point represents the OR for resistance to a specific antibiotic; the vertical line denotes OR = 1 (no association). None of the antibiotics, except the definitional β-lactams (Oxacillin and cefoxitin), showed statistically significant differences between MRSA and MSSA groups (*p* > 0.05)

MRSA isolates showed 100% resistance to Oxacillin and cefoxitin, confirming phenotypic resistance, while MSSA isolates remained fully susceptible to these agents. Overall resistance was highest for clindamycin (68.7%) and erythromycin (61.4%), followed by ciprofloxacin (41.0%) and meropenem 20.5%. Vancomycin resistance was detected in 5 isolates (6.0%) (Table 2). Comparison between MRSA and MSSA isolates revealed no statistically significant difference in resistance to meropenem, azithromycin, erythromycin, clindamycin, rifamycin, gentamicin, amikacin, or ciprofloxacin (all *p* > 0.05). Vancomycin resistance also showed no significant difference between the two groups (MRSA 7.9% vs. MSSA 4.4%, *p* = 0.31). In contrast, MDR was strongly associated with MRSA status. MRSA isolates exhibited nearly 5-fold higher odds of being multidrug resistant (OR = 4.9; 95% CI = 2.1–11.5; *p* = 0.001). Comparative odds ratios presented in Fig. 1.

**Table 2:** Antibiotic-specific resistance frequencies of *S. aureus* isolates (*n* = 83) and comparison between MRSA and MSSA groups

Antibiotic (µg)	Overall *R* / *N* (%)	MRSA *R* / *N* (%)	MSSA *R* / *N* (%)	Odds Ratio (95% CI)	*p* ^(Fisher/χ²)^
Oxacillin (1)	38 / 83 (45.8)	38 / 38 (100)	0 / 45 (0)	NA	NA
Cefoxitin (30)	38 / 83 (45.8)	38 / 38 (100)	0 / 45 (0)	NA	NA
Meropenem (10)	17 / 83 (20.5)	7 / 38 (18.4)	10 / 45 (22.2)	0.79 (0.28–2.19)	0.63
Azithromycin (15)	21 / 83 (25.3)	12 / 38 (31.6)	9 / 45 (20.0)	1.84 (0.68–4.95)	0.24
Erythromycin (15)	51 / 83 (61.4)	22 / 38 (57.9)	29 / 45 (64.4)	0.76 (0.32–1.80)	0.53
Clindamycin (2)	57 / 83 (68.7)	27 / 38 (71.1)	30 / 45 (66.7)	1.22 (0.48–3.13)	0.67
Rifampicin (5)	11 / 83 (13.3)	5 / 38 (13.2)	6 / 45 (13.3)	0.99 (0.27–3.68)	0.99
Gentamicin (10)	12 / 83 (14.5)	6 / 38 (15.8)	6 / 45 (13.3)	1.23 (0.35–4.37)	0.77
Amikacin (30)	16 / 83 (19.3)	7 / 38 (18.4)	9 / 45 (20.0)	0.90 (0.31–2.61)	0.85
Ciprofloxacin (5)	34 / 83 (41.0)	18 / 38 (47.4)	16 / 45 (35.6)	1.63 (0.67–3.93)	0.28
Vancomycin	5 / 83 (6.0)	3 / 38 (7.9)	2 / 45 (4.4)	1.84 (0.29–11.7)	0.31

NA indicates not applicable, as oxacillin and cefoxitin were used for phenotypic definition of MRSA and MSSA, and odds ratios and p-values were therefore not calculated for these antibiotics

### Distribution of MDR and XDR isolates

Based on the criteria of Magiorakos et al. (2012) [25], 55 isolates (66.3%) were identified as MDR, and two isolates (2.4%) were detected as XDR (Table 3). The predominant MDR pattern involved β-lactam, macrolide, and lincosamide resistance, particularly among MRSA isolates.

MRSA was strongly associated with MDR (OR = 4.9; 95% CI = 2.1–11.5; *p* = 0.001). MRSA showed higher odds of macrolide resistance (OR = 1.84), although this result was not statistically significant (*p* = 0.24).

**Table 3:** Distribution of multidrug-resistant (MDR) and extensive drug-resistant (XDR) *Staphylococcus aureus* isolates by antibiotic class (*n* = 83)

Antibiotic class	Antibiotics included	MDR isolates (*n* = 55)	% within class	XDR isolates (*n* = 2)	% within class	Odds Ratio (OR)	*p*-Value
β-lactams	Oxacillin, Cefoxitin, Meropenem	55	100.0	2	100.0	4.9	0.001
Macrolides	Azithromycin, Erythromycin	49	89.1	2	100.0	1.84	0.24
Lincosamide	Clindamycin	41	74.5	2	100.0	1.22	0.67
Aminoglycosides	Gentamicin, Amikacin	32	58.2	2	100.0	0.90	0.85
Fluoroquinolone	Ciprofloxacin	27	49.1	2	100.0	1.63	0.28
Ansamycin	Rifampicin	11	20.0	0	0.0	0.99	0.99
Glycopeptide	Vancomycin	5	9.1	0	0.0	1.84	0.31

### Multiple antibiotic resistance (MAR) index

The MAR index among *S. aureus* isolates ranged from 0.08 to 0.75(mean ± SD of 0.38 ± 0.12). High-risk MAR (≥ 0.2) occurred in 72.3% of isolates, and very high MAR (≥ 0.3) in 48.2% (Table 4). MRSA isolates showed significantly higher MAR values than MSSA (median 0.45 vs. 0.29; Mann-Whitney U = 255; *p* = 0.003). Very high MAR (≥ 0.3) was more frequent in (63.2%) than (35.5%) (*p* = 0.0011). Detailed values are presented in Table 4 and Supplementary Table 1.

**Table 4:** Multiple antibiotic resistance (MAR) index values and comparison between MRSA and MSSA isolates

Group	Range	Mean ± SD	Median (IQR)	Mann-Whitney U	*p*-value	Effect size (rank-biserial *r*)
MRSA (*n* = 38)	0.22–0.75	0.45 ± 0.10	0.45 (0.38–0.53)	255	0.003	0.42
MSSA (*n* = 45)	0.08–0.52	0.31 ± 0.11	0.29 (0.21–0.36)	-	-	-
Overall (*n* = 83)	0.08–0.75	0.38 ± 0.12	0.37 (0.26–0.46)	-	-	-

### Co-resistance and pattern variation

**Fig. 2:**
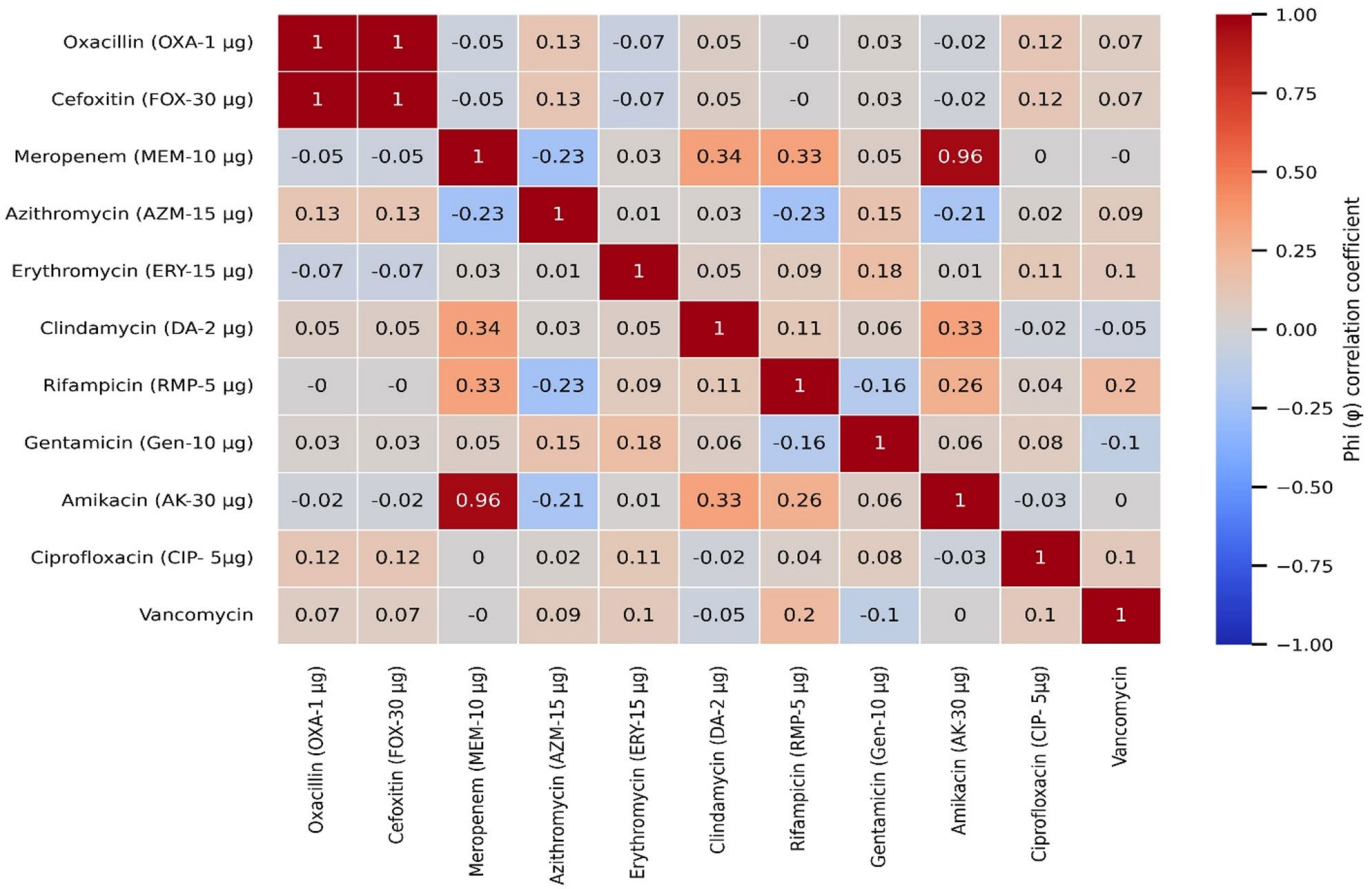
Phi (φ) correlation heatmap of antibiotic co-resistance. Indicating the strength and direction of their co-resistance relationship, red shades indicate positive co-resistance relationships, while blue shades reflect negative or independent associations. Strong correlations highlight antibiotics that share similar resistance patterns

Hierarchical clustering identified two major resistance clusters. One cluster showed a distinct β-lactam resistance pattern, characterized by strong co-resistance between Oxacillin and cefoxitin. The second cluster primarily comprised macrolide lincosamide resistance, involving erythromycin and clindamycin (Supplementary Fig. 2). These patterns were supported by the phi (φ) correlation analysis (Fig. 2). Oxacillin and cefoxitin showed perfect co-resistance (φ = 1.00), while meropenem and amikacin demonstrated a strong association (φ = 0.96). Lastly, Meropenem also demonstrated moderate correlations with clindamycin (φ = 0.34) and rifampicin (φ = 0.33), whereas azithromycin exhibited a weak negative correlation with Meropenem (φ = -0.23).

### MAR index variation across sampling areas and environments

MAR values varied across sampling locations (Supplementary Fig. 3A). MAR ranges from 0.29 ± 0.07 to 0.52 ± 0.10. Highest values were observed in Mirpur, Khilkhet, and Airport zones, while Banani and Gulshan recorded lower values. However, we did not identify statistically significant differences among areas (H = 8.88, *p* = 0.261). Higher resistance was observed in densely populated and low-sanitation areas. Based on the environmental groups, results revealed similar patterns (Supplementary Fig. 3B) with the mean MAR ranging from 0.31 ± 0.06 (Educational Area) to 0.53 ± 0.09 (Slum). The Kruskal-Wallis test also showed no significant differences (H = 2.89, *p* = 0.823). Higher MAR values were consistently observed in slum and transport hub areas. These findings suggest an association between higher MAR values and high traffic, low sanitation environments. Detailed data are provided in Supplementary Fig. 3 and Supplementary Table 2.

## Discussion

The overall prevalence of *S. aureus* (79.8%) and MRSA isolates (45.8%) indicated a substantial level of contamination within this informal food sector. To date, no study has specifically reported such contamination in street-vended tomato sauces in Bangladesh. However, comparable MRSA prevalence has been reported in ready-to-eat foods in Bangladesh and India [18, 27]. One study conducted in India identified 52 (12.1%) *S. aureus* isolated from street-vended foods, with resistance rates 36.5% for oxacillin, 25% for cefoxitin, and 82.7% penicillin G [28]. In comparison, we identified a higher proportion of resistant isolates, with 66.3% exhibiting MDR, 2.4% XDR, and two isolates exhibiting VRSA. Another study from Bangladesh reported a lower prevalence (22%) of *S. aureus* in raw and ready-to- cook foods [29]. However, High MDR rates (72.4%) in foodborne pathogens have also been reported in Bangladesh [30]. Internationally, MRSA prevalence has been reported at 52.8% in China [31] and 50.7% Egypt in food-related samples [32]. The result of vancomycin non-susceptible isolates is concerning giving the clinical importance of vancomycin in MRSA treatment [20]. However, these findings are based on phenotypic screening and may not necessarily indicate clinical treatment failure. The findings of the MAR index among the *S. aureus* isolates ranged from 0.08 to 0.75 (mean of 0.38 ± 0.12), with approximately 72% of isolates exceeding the high-risk threshold (≥ 0.2), indicating exposure to antibiotic-influenced environments [26]. Comparable MAR patterns have been reported in street food studies from different regions of the world [4, 14, 27, 33–37]. Co-resistance analysis further demonstrated strong associations between oxacillin-cefoxitin and meropenem-amikacin, suggesting linked resistance mechanisms across antibiotic classes. Similar associations have been reported, where mobile genetic elements such as plasmids and integrons facilitate the co-transfer of resistance genes [38, 39]. One research study with 1,611 Staphylococcus aureus isolates showed the correlation of oxacillin (1 µg) and cefoxitin (30 µg) for detecting mecA-mediated resistance in *Staphylococcus aureus* [40]. Supporting the observed resistance pattern in this study, geographical variation indicated higher MAR values in isolates from slum and transport hub areas compared to residential or institutional zones [41]. Informal production practices and limited regulatory oversight may contribute to microbiological contamination [11, 12]. In Bangladesh, studies have reported contamination of food products, including tomato-based items, with pathogens such as Enterobacteriaceae, *E*. *coli*, *Vibrio cholerae*, and *Salmonella*, along with a high proportion of MDR isolates [30, 42]. Tomato-based sauces are particularly susceptible to bacterial growth due to their high moisture content. In informal settings, inadequate preservation and hygiene practices during handling and serving may further increase contamination risk [8]. The detection of MRSA, MDR, and VRSA phenotypes in this study highlights the potential role of street-vended sauces as reservoirs for antimicrobial-resistant *S. aureus* within the community. Higher resistance observed in high traffic and low sanitation area in consistent with the previous findings [43, 44], suggesting that environmental exposure and handling practices may influence resistance patterns.

## Conclusion

This study identified a high prevalence of *Staphylococcus aureus* contamination in street-vended tomato sauces collected from different areas of Dhaka, including WHO-prioritized resistant strains such as MRSA, MDR, and occasional VRSA. Elevated MAR index values suggest exposure to an antibiotic-influenced environment. These findings highlight the potential role of widely consumed condiments in the transmission of antimicrobial-resistant S. aureus and underscore the need for improved vendor hygiene, safe food-handling practices, and routine monitoring to reduce foodborne and public health risks.

## Limitations

This study has several limitations. Firstly, MRSA and VRSA identification was based solely on phenotypic methods (disk diffusion) without molecular confirmation (e.g., PCR for *nuc*,* mecA*,* mecC*,* vanA*,* vanB*) or MIC testing for vancomycin. Second, quantitative enumeration of *Staphylococcus aureus* was not performed, and the contamination levels (bacterial load) could not be assessed. Third, enterotoxin genes were not evaluated, limiting the assessment of the pathogenic potential of the isolates. Additionally, Convenience sampling and the collection of a single sample per vendor may not fully represent all street food vendors. The geographic scope was limited to Dhaka, Bangladesh, and non-standardized collection timing limits inference on temporal variability. Future studies will include molecular confirmation, quantitative microbial analysis, toxic gene detection and border sampling strategies to strengthen risk assessment.

## Supplementary Information

Below is the link to the electronic supplementary material.

Supplementary Material 1.

## Data Availability

All datasets and supporting laboratory findings used in this study have been fully included in the manuscripts.

## References

[1] Food safety. https://www.who.int/news-room/fact-sheets/detail/food-safety Accessed 21 Oct 2025

[2] Tibebu A, Tamrat H, Bahiru A, Review. Impact of food safety on global trade. Vet Med Sci [Internet]. 2024;10(5):e1585. Available from: http://www.ncbi.nlm.nih.gov/pubmed/39158975 10.1002/vms3.1585 PMC11332392 39158975

[3] Kamala K, Kumar VP. Food Products and Food Contamination. In: Microbial Contamination and Food Degradation. Elsevier; 2018. pp. 1–19. https://linkinghub.elsevier.com/retrieve/pii/B9780128115152000019

[4] Makhunga S, Hlongwana K. Food handling practices and sanitary conditions of charitable food assistance programs in eThekwini District, KwaZulu-Natal, South Africa. Sci Rep. 2024;14(1):26366. https://www.nature.com/articles/s41598-024-72359-2 10.1038/s41598-024-72359-2 PMC11530687 39487140

[5] Islam S, Tanjia N, Mitra AK, Hossain SJ, Hossain A, Jasika MT et al. Lack of Food Safety and Hygienic Practices among Street Vendors in Dhaka, Bangladesh: Implications for Consumers’ Health. 2023. https://www.preprints.org/manuscript/202307.0344/v1

[6] Food-Safety-Act. -2013 - বাংলাদেশ নিরাপদ খাদ্য কর্তৃপক্ষ- . [Accessed 21 Oct 2025]. https://bfsa.gov.bd/site/view/law/Food-Safety-Act ,-2013.

[7] Al Mamun M, Rahman SMM, Turin TC. Microbiological quality of selected street food items vended by school-based street food vendors in Dhaka, Bangladesh. Int J Food Microbiol. 2013;166(3):413–8. https://linkinghub.elsevier.com/retrieve/pii/S0168160513003814 10.1016/j.ijfoodmicro.2013.08.007 24029025

[8] Islam S, Nasrin N, Rizwan F, Nahar L, Bhowmik A, Esha SA et al. Microbial contamination of street vended foods from a university campus in bangladesh. Southeast Asian J Trop Med Public Health 2015;46(3):480–5. http://www.ncbi.nlm.nih.gov/pubmed/26521522 26521522

[9] Wang C, Li M, Duan X, Abu-Izneid T, Rauf A, Khan Z et al. Phytochemical and Nutritional Profiling of Tomatoes; Impact of Processing on Bioavailability - A Comprehensive Review. Food Rev Int. 2023;39(8):5986–6010. https://www.tandfonline.com/doi/full/10.1080/87559129.2022.2097692

[10] Anandsynal, Mumtaz B, Motalab M, Jahan S, Hoque M, Saha B. Nutritional and microbiological evaluation on sauces and ketchups available in Bangladesh. Int Food Res J. 2018;25:357–65.

[11] Mehrin S, Zakir HM, Seal HP, Akter M. Nutritional Quality and Metallic Health Risk Assessment of Industrially Processed Tomato Ketchups Available in the Markets of Bangladesh. Eur J Nutr Food Saf. 2020;67–78. https://journalejnfs.com/index.php/EJNFS/article/view/422

[12] Boakye A, Avor DD, Amponsah IK, Appaw WO, Owusu-Ansah L, Adjei S et al. Quality Assessment of Tomato Paste Products on the Ghanaian Market: An Insight Into Their Possible Adulteration. Chandrasekar CM, editor. Int J Food Sci. 2024;2024(1). https://onlinelibrary.wiley.com/doi/10.1155/2024/8285434 10.1155/2024/8285434 PMC11405106 39285917

[13] Sati H, Carrara E, Savoldi A, Hansen P, Garlasco J, Campagnaro E, et al. The WHO Bacterial Priority Pathogens List 2024: a prioritisation study to guide research, development, and public health strategies against antimicrobial resistance. Lancet Infect Dis. 2025;25(9):1033–43. 40245910 10.1016/S1473-3099(25)00118-5 PMC12367593

[14] Wang W, Baloch Z, Jiang T, Zhang C, Peng Z, Li F et al. Enterotoxigenicity and Antimicrobial Resistance of Staphylococcus aureus Isolated from Retail Food in China. Front Microbiol. 2017;8. http://journal.frontiersin.org/article/10.3389/fmicb.2017.02256/full 10.3389/fmicb.2017.02256 PMC5702451 29209290

[15] Oniciuc EA, Nicolau AI, Hernández M, Rodríguez-Lázaro D. Presence of methicillin-resistant Staphylococcus aureus in the food chain. Trends Food Sci Technol. 2017;61:49–59. https://linkinghub.elsevier.com/retrieve/pii/S0924224416304666

[16] Kluytmans JAJW. Methicillin-resistant Staphylococcus aureus in food products: cause for concern or case for complacency? Clin Microbiol Infect. 2010;16(1):11–5. https://linkinghub.elsevier.com/retrieve/pii/S1198743X14617778 10.1111/j.1469-0691.2009.03110.x 20002686

[17] Elsalkh SA, Zakaria AI, Abd-Elghany SM, Imre K, Morar A, Sallam KI. Prevalence and Characterization of the Antimicrobial Resistance and Virulence Profiles of Staphylococcus aureus in Ready-to-Eat (Meat, Chicken, and Tuna) Pizzas in Mansoura City, Egypt. Antibiotics. 2025;14(8):817. https://www.mdpi.com/2079-6382/14/8/817 10.3390/antibiotics14080817 PMC12382634 40868011

[18] Ali Alghamdi B, Al-Johani I, Al-Shamrani JM, Musamed Alshamrani H, Al-Otaibi BG, Almazmomi K et al. Antimicrobial resistance in methicillin-resistant staphylococcus aureus. Saudi J Biol Sci. 2023;30(4):103604. https://linkinghub.elsevier.com/retrieve/pii/S1319562X23000499 10.1016/j.sjbs.2023.103604 PMC10018568 36936699

[19] Samtiya M, Matthews KR, Dhewa T, Puniya AK. Antimicrobial Resistance in the Food Chain: Trends, Mechanisms, Pathways, and Possible Regulation Strategies. Foods. 2022;11(19):2966. https://www.mdpi.com/2304-8158/11/19/2966 10.3390/foods11192966 PMC9614604 36230040

[20] McGuinness WA, Malachowa N, DeLeo FR. Vancomycin Resistance in Staphylococcus aureus. Yale J Biol Med. 2017;90(2):269–81. http://www.ncbi.nlm.nih.gov/pubmed/28656013 PMC5482303 28656013

[21] Haque MA, Hu H, Liu J, Islam MA, Hossen F, Rahman MA, et al. Emergence of multidrug-resistant Bacillus spp. derived from animal feed, food and human diarrhea in South-Eastern Bangladesh. BMC Microbiol. 2024;24(1):61. 38373893 10.1186/s12866-024-03199-3 PMC10875756

[22] Hossain FE, Islam S, Islam MA, Islam S, Ahmed F. Detection of virulence genes of APEC (avian pathogenic Escherichia coli) isolated from poultry in Noakhali, Bangladesh. Bioresearch Commun. 2021;7(1):967–72.

[23] Trujillo ME, Dedysh S, DeVos P, Hedlund B, Kämpfer P, Rainey FA, et al. editors. Bergey’s Manual of Systematics of Archaea and Bacteria. Wiley; 2015.

[24] Rai S, Dash D, Agarwal N. Introducing the new face of CLSI M100 in 2023: An explanatory review. Indian J Med Microbiol [Internet]. 2023;46:100432. Available from: https://linkinghub.elsevier.com/retrieve/pii/S0255085723001482 10.1016/j.ijmmb.2023.100432 37945125

[25] Magiorakos AP, Srinivasan A, Carey RB, Carmeli Y, Falagas ME, Giske CG et al. Multidrug-resistant, extensively drug-resistant and pandrug-resistant bacteria: an international expert proposal for interim standard definitions for acquired resistance. Clin Microbiol Infect [Internet]. 2012;18(3):268–81. Available from: https://linkinghub.elsevier.com/retrieve/pii/S1198743X14616323 10.1111/j.1469-0691.2011.03570.x 21793988

[26] Krumperman PH. Multiple antibiotic resistance indexing of Escherichia coli to identify high-risk sources of fecal contamination of foods. Appl Environ Microbiol [Internet]. 1983;46(1):165–70. Available from: https://journals.asm.org/doi/10.1128/aem.46.1.165-170.1983 10.1128/aem.46.1.165-170.1983 PMC239283 6351743

[27] Ghosh M, Wahi S, Kumar M, Ganguli A. Prevalence of enterotoxigenic Staphylococcus aureus and Shigella spp. in some raw street vended Indian foods. Int J Environ Health Res [Internet]. 2007;17(2):151–6. Available from: http://www.tandfonline.com/doi/abs/10.1080/09603120701219204 10.1080/09603120701219204 17616871

[28] Sivakumar M, Dubal ZB, Kumar A, Bhilegaonkar K, Vinodh Kumar OR, Kumar S, et al. Virulent methicillin resistant Staphylococcus aureus (MRSA) in street vended foods. J Food Sci Technol. 2019;56(3):1116–26. 30956291 10.1007/s13197-019-03572-5 PMC6423183

[29] Islam MA, Parveen S, Rahman M, Huq M, Nabi A, Khan ZUM et al. Occurrence and Characterization of Methicillin Resistant Staphylococcus aureus in Processed Raw Foods and Ready-to-Eat Foods in an Urban Setting of a Developing Country. Front Microbiol. 2019;10. 10.3389/fmicb.2019.00503 PMC6426745 30923520

[30] Samad MA, Eberson L, Begum R, Alam MGS, Talukdar F, Akter R, et al. Microbial Contamination and Antibiotic Resistance in Marketed Food in Bangladesh: Current Situation and Possible Improvements. Antibiotics. 2023;12(3):555. 36978422 10.3390/antibiotics12030555 PMC10044357

[31] Wu S, Huang J, Zhang F, Wu Q, Zhang J, Pang R et al. Prevalence and Characterization of Food-Related Methicillin-Resistant Staphylococcus aureus (MRSA) in China. Front Microbiol. 2019;10. 10.3389/fmicb.2019.00304 PMC6391343 30842766

[32] Elsalkh SA, Zakaria AI, Abd-Elghany SM, Imre K, Morar A, Sallam KI. Prevalence and Characterization of the Antimicrobial Resistance and Virulence Profiles of Staphylococcus aureus in Ready-to-Eat (Meat, Chicken, and Tuna) Pizzas in Mansoura City. Egypt Antibiot. 2025;14(8):817. 10.3390/antibiotics14080817 PMC12382634 40868011

[33] Moges M, Rodland EK, Legesse T, Argaw A. Antibiotic resistance patterns of Staphylococcus aureus and Enterobacteriaceae isolated from street foods in selected towns of Ethiopia. BMC Infect Dis [Internet]. 2024;24(1):367. Available from: https://bmcinfectdis.biomedcentral.com/articles/10.1186/s12879-024-09266-4 10.1186/s12879-024-09266-4 PMC10986114 38566010

[34] Fusaro C, Miranda-Madera V, Serrano-Silva N, Bernal JE, Ríos-Montes K, González-Jiménez FE et al. Antibiotic-Resistant Bacteria Isolated from Street Foods: A Systematic Review. Antibiotics [Internet]. 2024;13(6):481. Available from: https://www.mdpi.com/2079-6382/13/6/481 10.3390/antibiotics13060481 PMC11201236 38927148

[35] Sharif N, Opu RR, Saha T, Khan A, Alzahrani FM, Alsuwat MA et al. Antimicrobial resistant enteric bacteria are widely distributed among environmental water sources in Dhaka, Bangladesh. npj Clean Water [Internet]. 2025;8(1):16. Available from: https://www.nature.com/articles/s41545-025-00447-5

[36] Igbinosa EO, Beshiru A, Igbinosa IH, Ogofure AG, Ekundayo TC, Okoh AI. Prevalence, multiple antibiotic resistance and virulence profile of methicillin-resistant Staphylococcus aureus (MRSA) in retail poultry meat from Edo, Nigeria. Front Cell Infect Microbiol. 2023;13. 10.3389/fcimb.2023.1122059 PMC10017849 36936767

[37] Letuka P, Nkhebenyane SJ, Ramatla T, Zikhona TN, Lekota KE, Khasapane NG. Antimicrobial resistance and genomic characterization of Staphylococcus aureus in ready-to-eat foods from Mangaung Metro Municipality. Front Microbiol. 2025;16. 10.3389/fmicb.2025.1669035 PMC12714933 41425926

[38] Sajerli B, Makai K, Lakatos L, Sarkadi-Nagy Á, Burián K, Orosz L. Aminoglycoside resistance dynamics and its predictive value for carbapenem resistance in multidrug-resistant Acinetobacter baumannii and Klebsiella pneumoniae. J Glob Antimicrob Resist [Internet]. 2025;43:309–18. Available from: https://linkinghub.elsevier.com/retrieve/pii/S2213716525001183 10.1016/j.jgar.2025.05.012 40409495

[39] Aminov RI. A Brief History of the Antibiotic Era: Lessons Learned and Challenges for the Future. Front Microbiol. 2010;1. 10.3389/fmicb.2010.00134 PMC3109405 21687759

[40] Broekema NM, Van TT, Monson TA, Marshall SA, Warshauer DM. Comparison of Cefoxitin and Oxacillin Disk Diffusion Methods for Detection of mecA -Mediated Resistance in Staphylococcus aureus in a Large-Scale Study. J Clin Microbiol. 2009;47(1):217–9. 19020073 10.1128/JCM.01506-08 PMC2620872

[41] Shafique S, Bhattacharyya DS, Nowrin I, Sultana F, Islam MR, Dutta GK, et al. Effective community-based interventions to prevent and control infectious diseases in urban informal settlements in low- and middle-income countries: a systematic review. Syst Rev. 2024;13(1):253. 39367477 10.1186/s13643-024-02651-9 PMC11451040

[42] Hossain M, Dey BK. Microbial Contamination of Handmade Sauce Used by Street Food Vendors in Jashore, Bangladesh. J Food Qual Hazards Control. 2019.

[43] Egwu KC, Abdulkarim M, Adebisi YA, Cariaga MFN. Antimicrobial Resistance in Slums: A Call for Global Action. Public Heal Challenges [Internet]. 2025;4(1). Available from: https://onlinelibrary.wiley.com/doi/10.1002/puh2.70028 10.1002/puh2.70028 PMC12039356 40496117

[44] Vassallo A, Kett S, Purchase D, Marvasi M. The Bacterial Urban Resistome: Recent Advances. Antibiotics. 2022;11(4):512. https://www.mdpi.com/2079-6382/11/4/512 10.3390/antibiotics11040512 PMC9030810 35453263

