# Supplementary material for: Prevalence of methicillin-resistant Staphylococcus aureus (MRSA) in street-vended tomato sauces in Dhaka, Bangladesh
Source: BMC Res Notes. 2026 May 9;19:269. doi: 10.1186/s13104-026-07822-6 (PMC13326474; doi:10.1186/s13104-026-07822-6)
Supplement: Supplementary file 1 — Supplementary Material 1. [file 13104_2026_7822_MOESM1_ESM.zip › Supplementary/Supplementary Figure 1.docx]

**
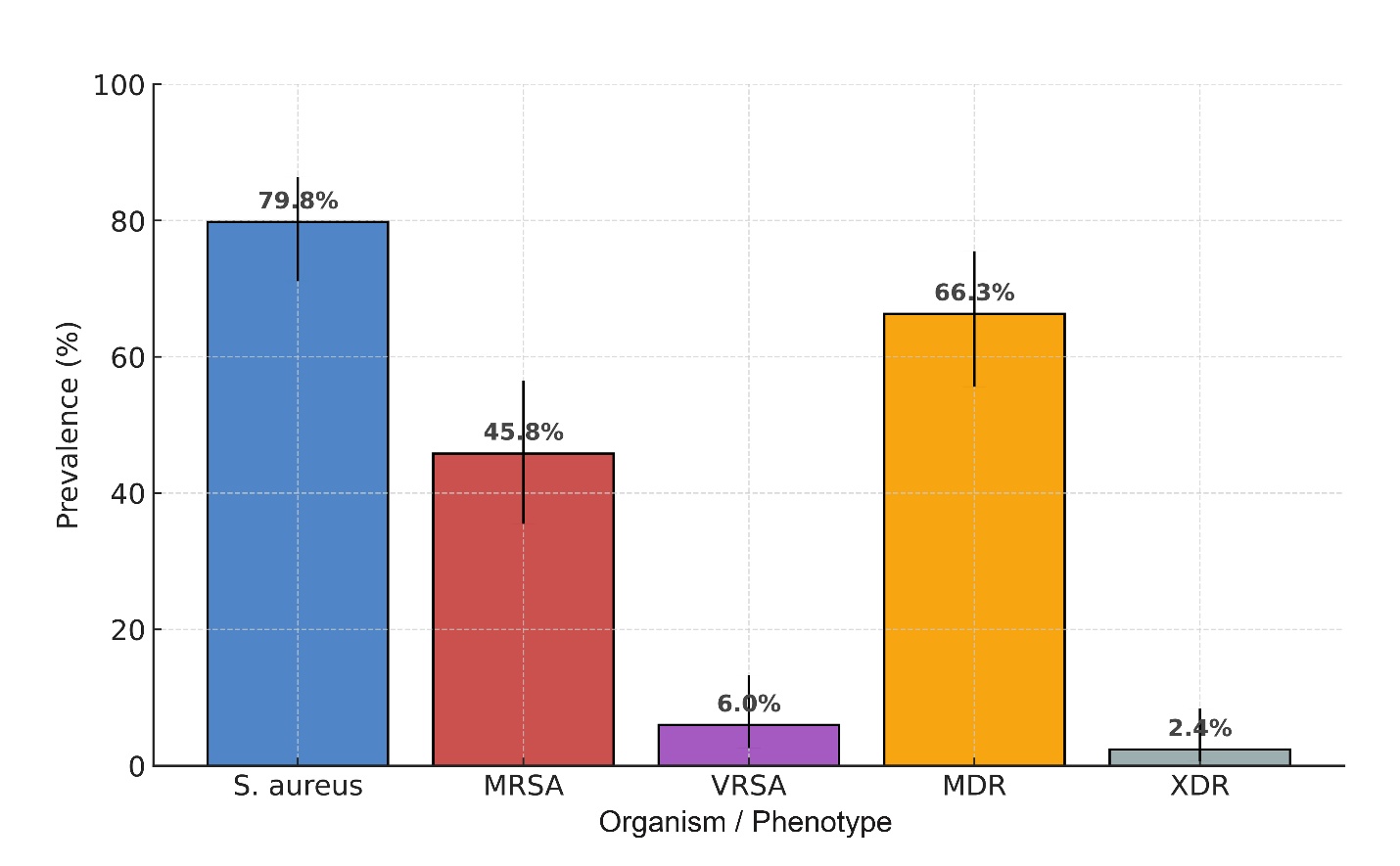
**

**Supplementary Figure 1. Prevalence of *S. aureus* and major antibiotic-resistant phenotypes in tomato sauces(n=83),** *Error bars represent Wilson 95% Cls.*
