# Supplementary material for: Prevalence of methicillin-resistant Staphylococcus aureus (MRSA) in street-vended tomato sauces in Dhaka, Bangladesh
Source: BMC Res Notes. 2026 May 9;19:269. doi: 10.1186/s13104-026-07822-6 (PMC13326474; doi:10.1186/s13104-026-07822-6)
Supplement: Supplementary file 1 — Supplementary Material 1. [file 13104_2026_7822_MOESM1_ESM.zip › Supplementary/Supplementary Figure 2.docx]

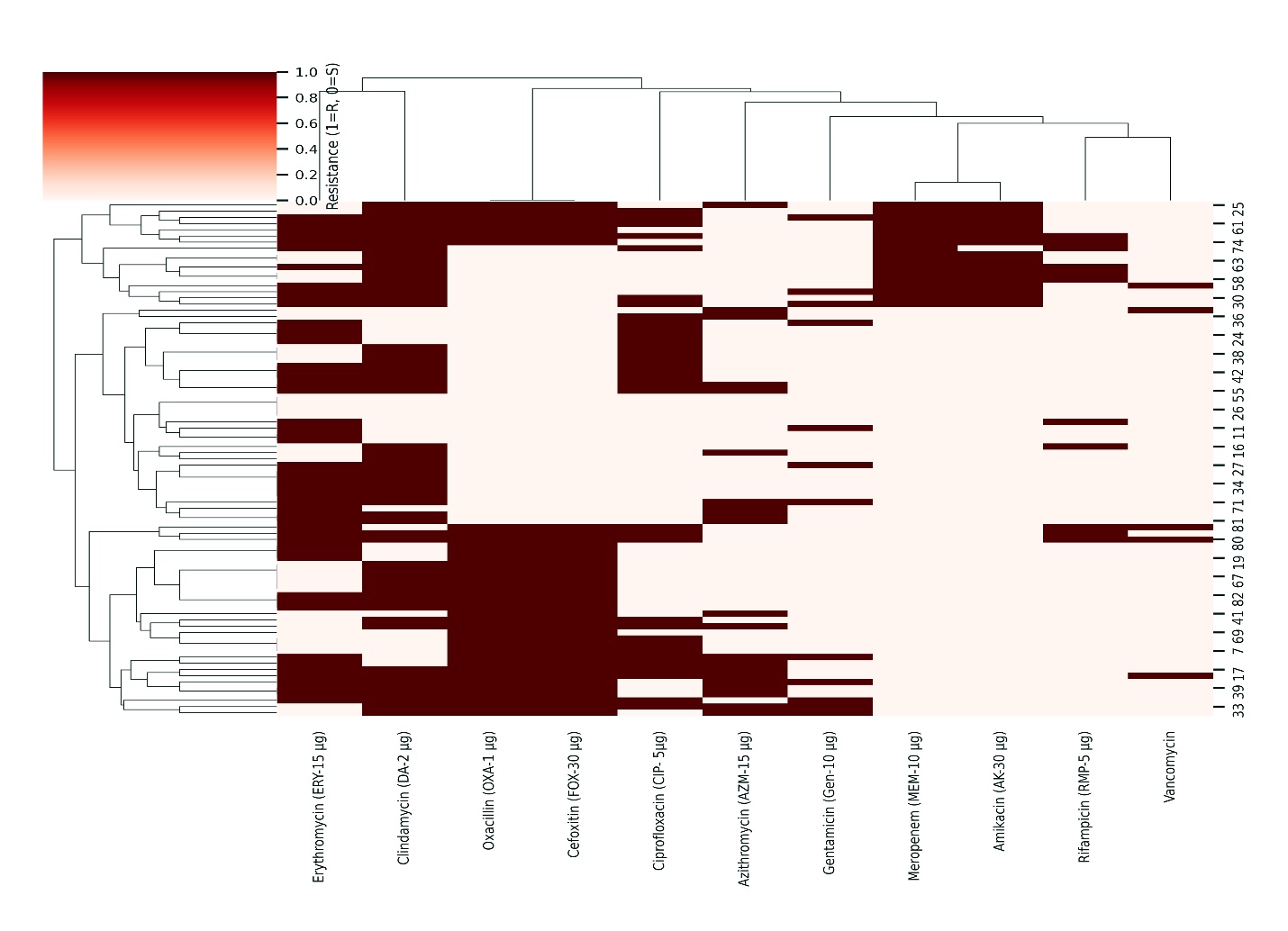


**Supplementary Figure 2. Hierarchical heatmap of Co-resistance patterns among 83 Staphylococcus aureus isolated from tomato sauces.** *Red cells indicate resistant responses(R=1) and white cells indicate sensitivity (S=0). Isolates (rows) and antibiotics (columns) were clustered using Euclidean distance average linkage. Two prominent resistance clusters were evident β-lactams group and the macrolide lincosamide group, and other antibiotics exhibiting variable or isolated resistance profiles.*
