# Supplementary material for: Prevalence of methicillin-resistant Staphylococcus aureus (MRSA) in street-vended tomato sauces in Dhaka, Bangladesh
Source: BMC Res Notes. 2026 May 9;19:269. doi: 10.1186/s13104-026-07822-6 (PMC13326474; doi:10.1186/s13104-026-07822-6)
Supplement: Supplementary file 1 — Supplementary Material 1. [file 13104_2026_7822_MOESM1_ESM.zip › Supplementary/Supplementary Figure 3A B.docx]

**
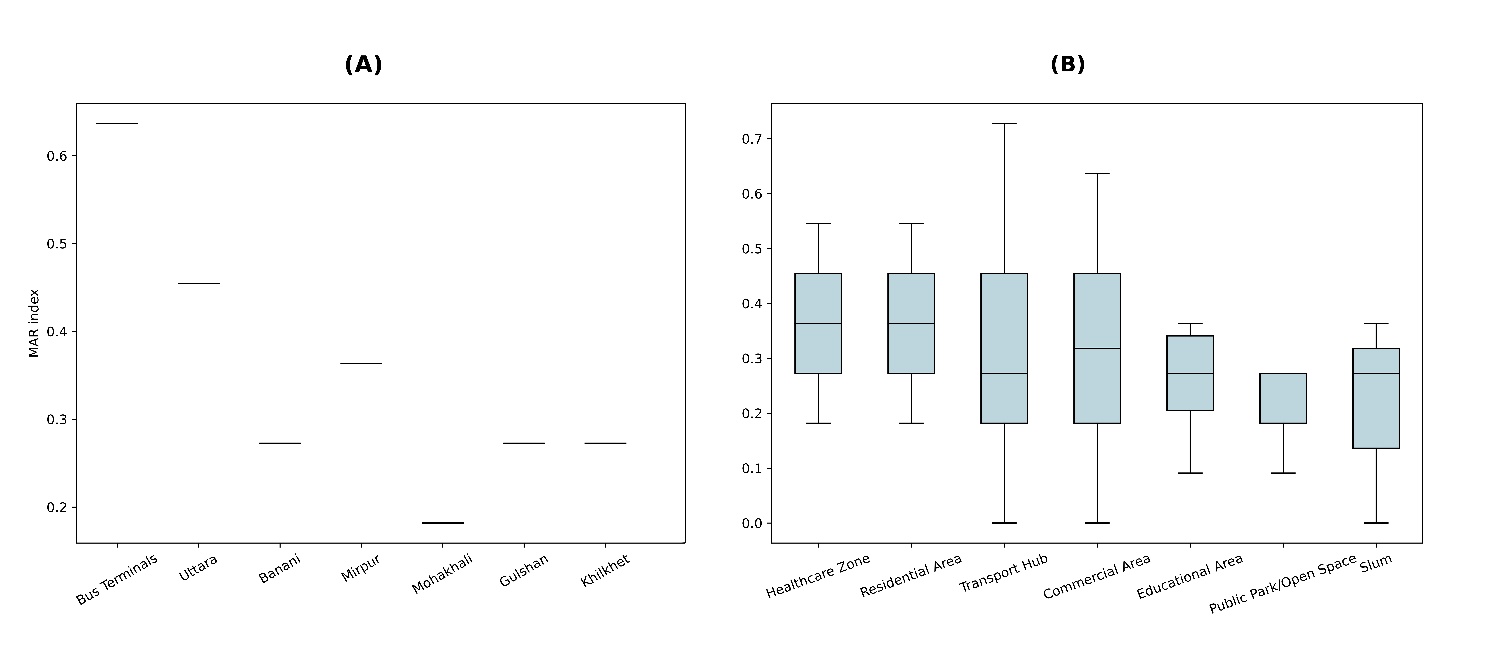
**

**Supplementary Figure 3.** **Variation in Multiple Antibiotic Resistance (MAR) index among *S. aureus* isolates from tomato sauces in Dhaka***. (A) MAR index by sampling Area. (B) MAR index by environmental category (Transport Hub, Slum, Residential Area, Healthcare Zone, Educational Area, Commercial Area, and Public Park/Open Space). Boxes represent interquartile ranges, medians are shown as horizontal lines, and whiskers denote the full range of values*.
