# Supplementary material for: Prevalence of methicillin-resistant Staphylococcus aureus (MRSA) in street-vended tomato sauces in Dhaka, Bangladesh
Source: BMC Res Notes. 2026 May 9;19:269. doi: 10.1186/s13104-026-07822-6 (PMC13326474; doi:10.1186/s13104-026-07822-6)
Supplement: Supplementary file 1 — Supplementary Material 1. [file 13104_2026_7822_MOESM1_ESM.zip › Supplementary/Supplementary Table 1.docx]

**Supplementary Table 1.** **Proportion of high-risk MAR isolates (≥ 0.2 and ≥ 0.3) and contingency-test comparison between MRSA and MSSA groups**

| **MAR threshold** | **MRSA (n = 38)** | **MSSA (n = 45)** | **Total (n = 83)** | **Test** | **p-value** | **Interpretation** |
| --- | --- | --- | --- | --- | --- | --- |
| ≥ 0.2 (high-risk) | 33 (86.8 %) | 27 (60.0 %) | 60 (72.3 %) | χ² / Fisher | 0.004 | Significant association - high-MAR phenotypes enriched in MRSA |
| ≥ 0.3 (very high-risk) | 24 (63.2 %) | 16 (35.5 %) | 40 (48.2 %) | χ² / Fisher | 0.011 | Significant association: high MAR is more frequent in MRSA |
