# Supplementary material for: Prevalence of methicillin-resistant Staphylococcus aureus (MRSA) in street-vended tomato sauces in Dhaka, Bangladesh
Source: BMC Res Notes. 2026 May 9;19:269. doi: 10.1186/s13104-026-07822-6 (PMC13326474; doi:10.1186/s13104-026-07822-6)
Supplement: Supplementary file 1 — Supplementary Material 1. [file 13104_2026_7822_MOESM1_ESM.zip › Supplementary/Supplementary Table 2 MAR Distribution.docx]

**Supplementary Table 2 MAR Distribution**

| **Group Type** | **Category** | **n** | **MAR Mean ± SD** | **Min** | **Max** | **Rank** |
| --- | --- | --- | --- | --- | --- | --- |
| Area | Banani | 1 | 0.273 ± nan | 0.273 | 0.273 | 5 |
| Area | Bus Terminals | 1 | 0.636 ± nan | 0.636 | 0.636 | 1 |
| Area | Gulshan | 1 | 0.273 ± nan | 0.273 | 0.273 | 5 |
| Area | Khilkhet | 1 | 0.273 ± nan | 0.273 | 0.273 | 5 |
| Area | Mirpur | 1 | 0.364 ± nan | 0.364 | 0.364 | 3 |
| Area | Mohakhali | 1 | 0.182 ± nan | 0.182 | 0.182 | 7 |
| Area | Uttara | 1 | 0.455 ± nan | 0.455 | 0.455 | 2 |
| Env_Group | Commercial Area | 46 | 0.332 ± 0.184 | 0 | 0.727 | 3 |
| Env_Group | Educational Area | 4 | 0.25 ± 0.114 | 0.091 | 0.364 | 5 |
| Env_Group | Healthcare Zone | 2 | 0.364 ± 0.257 | 0.182 | 0.545 | 1 |
| Env_Group | Public Park/Open Space | 5 | 0.218 ± 0.081 | 0.091 | 0.273 | 6 |
| Env_Group | Residential Area | 2 | 0.364 ± 0.257 | 0.182 | 0.545 | 1 |
| Env_Group | Slum | 3 | 0.212 ± 0.189 | 0 | 0.364 | 7 |
| Env_Group | Transport Hub | 28 | 0.312 ± 0.189 | 0 | 0.727 | 4 |
